# Evidence-based public health messaging on the non-visual effects of ocular light exposure: A modified Delphi expert consensus

**DOI:** 10.1101/2025.05.07.25327160

**Published:** 2025-05-08

**Authors:** Manuel Spitschan, Laura Kervezee, Oliver Stefani, Marijke Gordijn, Jennifer A. Veitch, Renske Lok, On behalf of the Ladenburg Light and Health Consortium

**Affiliations:** 1 TUM School of Medicine & Health, Department of Health and Sport Sciences, Technical University of Munich, Munich, Germany; 2 TUM Institute for Advanced Study (TUM-IAS), Technical University of Munich, Garching, Germany; 3 Max Planck Institute for Biological Cybernetics, Max Planck Research Group Translational Sensory & Circadian Neuroscience, Tübingen, Germany; 4 TUMCREATE, Singapore, Singapore; 5 Department of Cellular and Chemical Biology, Leiden University Medical Center, Leiden, Netherlands; 6 Lucerne University of Applied Sciences and Arts, Lucerne, Switzerland; 7 Chrono@Work, Groningen, The Netherlands; 8 University of Groningen, Faculty of Science and Engineering, Groningen, The Netherlands; 9 National Research Council of Canada, Ottawa, Canada; 10 Department of Psychiatry and Behavioral Sciences, Stanford University, Stanford, USA

**Keywords:** Non-image-forming effects of light, Ocular light exposure, Health, Photobiology, Modified Delphi, Public engagement, Science communication, Policy translation

## Abstract

**Importance.:**

In addition to vision, light regulates circadian rhythms, sleep, mood and alertness. Despite growing scientific understanding, there remains a gap in translating this knowledge into accessible, evidence-based guidance for the public and policymakers.

**Objective.:**

To formulate scientifically grounded statements about the influence of light on human psychological and physiological health, intended for dissemination to the public and policymakers.

**Evidence Review.:**

An international consortium of 21 experts convened at the Ladenburg Roundtable in April 2024 to develop consensus-based public health messages on the physiological and psychological effects of light. Experts were selected based on their scientific contributions to chronobiology, psychology, neuroscience, and the measurement and practical application of light. Through a structured, iterative modified Delphi process involving multiple rounds of drafting, anonymous feedback, and revision, 27 statements were developed. Each statement included a simplified public-facing version and contextual information to support understanding. Consensus was assessed using predefined thresholds of agreement (>75% endorsement). Statements not meeting consensus were revised and re-evaluated.

**Findings.:**

Of the 27 proposed statements, 26 reached the threshold for consensus, with high levels of agreement across diverse topics, including the properties of light, its physiological and psychological effects, individual variability, and research gaps. One statement did not reach consensus due to insufficient scientific evidence and was excluded, while another was revised based on feedback and subsequently accepted. The iterative revision process significantly improved the clarity, accuracy, and accessibility of the final statements. A readability assessment showed an average sentence length of 14.8 words and a Flesch-Kincaid Grade Level of 8.6, indicating that the statements suit a broad, non-specialist audience. The final consensus statements are available at lightforpublichealth.org.

**Conclusions and Relevance.:**

This expert consensus provides clear, accessible messages about how light affects human health. By translating scientific evidence into understandable guidance, the statements offer a practical tool for public education and policymaking. The messages can be used by public-health multipliers (e.g., schools, employers, healthcare providers, and urban planners) to promote healthier light exposure in daily life. They highlight the importance of recognizing light as a key factor in health, alongside sleep, nutrition, and physical activity.

## Introduction

Over the past 50 years, the scientific understanding of how light affects human health has advanced dramatically. Beyond enabling vision, light regulates core biological processes, including circadian rhythms, sleep, and hormone secretion. The discovery of intrinsically photosensitive retinal ganglion cells (ipRGCs) revealed how light exerts effects on human physiology and psychology^1–3^. These cells play a central role in synchronizing the biological clock to the external light-dark cycle^4,5^, thereby influencing sleep-wake timing^6,7^. Additionally, light acutely affects alertness^8,9^, melatonin production (e.g ^9–11^), and cognitive performance^12,13^.

These discoveries prompted the development of standards for measuring biologically effective light. In 2018, the International Commission on Illumination introduced CIE S 026/E:2018, providing a standardized framework for quantifying biologically effective ocular light^14^. In 2022, global experts released recommendations for healthy light exposure^15^. However, the translation of these findings into public health guidance remains a challenge, hindered by a lag in the adoption of research findings in policy and public health guidelines^16^.

To address this gap, 21 international experts convened at the Ladenburg Roundtable (April 14–16, 2024, Germany) to develop consensus-based, evidence-informed statements on light and health. The goal was to support public health messaging, guide policy, inform architecture and workplace standards, and empower individuals. The resulting white paper identified seven key steps for effective communication, including simplifying technical language and aligning with existing public health recommendations^17^.

This report presents 26 consensus statements on the effects of light on human psychology and physiology, developed through an iterative review.

## Materials and Methods

### Participants

Participants for the Ladenburg Light & Health Roundtable were purposefully selected to ensure diversity in geography, career stage, and expertise. The final group included 25 experts from human clinical research, animal neuroscience, physiological optics, epidemiology, occupational health, environmental psychology, chronobiology, lighting science, and public health (Appendix A). They represented universities, research institutions, and public agencies. Four invited individuals were unable to attend. Of the 25 experts, 19 actively participated in the statement rating process. A coordinating committee of six lead authors representing five key organizations, the International Commission on Illumination (CIE), the Society for Light, Rhythms and Circadian Health (SLRCH), the Daylight Academy (DLA), the Good Light Group (GLG), and the Center for Environmental Therapeutics (CET), oversaw the process and endorsed the statements but did not contribute ratings. For this process, no public or patient groups were consulted in developing the consensus. Participants were not compensated for their participation. All invited experts completed the process.

### Statement development and consensus

**Figure 1.**
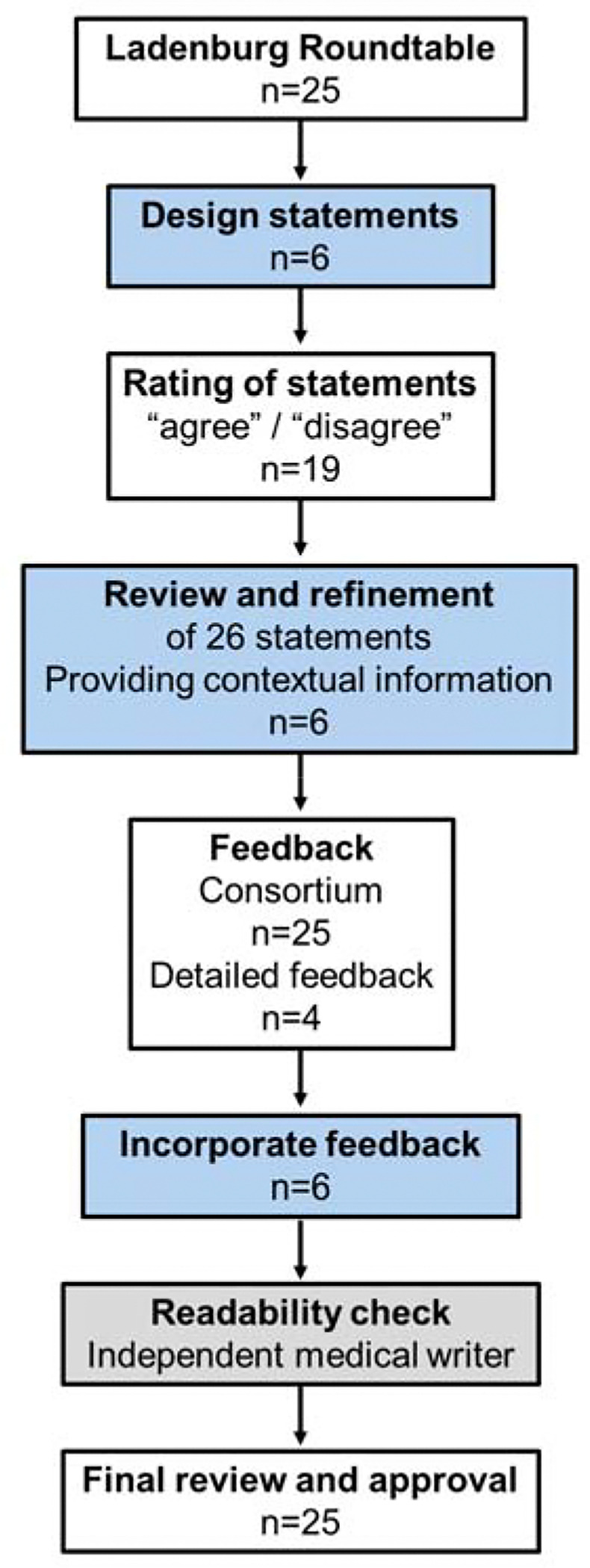
Flow chart. The coordinating committee led the initial development of key statements, which were then submitted for consensus approval by the Ladenburg Light & Health Consortium. Each statement received feedback, with in-depth input from four Consortium members incorporated into the revisions. Once the core statements were finalized, simplified versions and contextual information were developed, followed by an additional round of feedback. A medical writer then assessed the document for English readability, ensuring accessibility while preserving the original scientific statements. Finally, the Ladenburg Light & Health Consortium was given a final opportunity to review the scientific statements, along with their simplified versions and contextual details, before finalization. Boxes in light blue indicate steps led by the steering committee (n=6), white presents the Consortium (n=25) and grey the independent external medical writer (n=1).

We employed a modified Delphi technique involving two rounds of anonymized feedback using REDCap^18,19^, with a predefined consensus threshold of 75% agreement (Figure 1). The development was not registered. The coordinating committee developed the initial statements, incorporating feedback from roundtable discussions. No systematic searches were conducted. Committee members simplified language, added contextual information, and cited relevant evidence. A two-step peer-review process followed: in round one, participants rated and commented on the draft statements; feedback was integrated into revised versions. Participants reviewed simplified statements and contextual text in the second round, ensuring scientific accuracy and clarity. The entire process, including consensus-building, peer review, and readability adjustments, occurred between October 2024 and February 2025.

### Evidence rating

Each scientific statement developed in this consensus process was rated for evidence certainty following a structured approach. We based our assessment on a modified version of the Oxford Centre for Evidence-Based Medicine (OCEBM) Quality Rating Scheme for Studies and Other Evidence.

The certainty of evidence for each statement was assigned as follows:

- **Level 1 (High certainty)**: Properly powered and conducted randomized clinical trials, or systematic reviews with meta-analysis.
- **Level 2 (Moderate certainty)**: Well-designed controlled trials without randomization, or prospective comparative cohort studies.
- **Level 3 (Moderate to low certainty)**: Case-control studies or retrospective cohort studies.
- **Level 4 (Low certainty)**: Case series with or without intervention, or cross-sectional studies.
- **Level 5 (Very low certainty)**: Opinion of respected authorities, narrative reviews, case reports, or textbook knowledge.

### Readability rating

To support public understanding, a medical writer assessed readability and refined simplified statements to a high school reading level and key stakeholder groups, without altering the original scientific statements.

## Results

### Initial statement development

The 27 initial statements aimed to deliver clear, evidence-informed messages about the non-image-forming effects of light. They address a wide range of topics, including the properties of light, how the human eye detects and processes light, and the influence of light on various physiological and psychological functions, such as the regulation of circadian rhythms, sleep, mood, and alertness. The statements also consider how these responses may change with age and include reflections on the current state of research in the field.

A consensus threshold of over 75% agreement was met for all but two of the original statements:

1. “Light exposure can be described by its intensity: The total amount of energy across all wavelengths from 380 to 780 nm.”
2. “Higher light levels during the daytime reduce the physiological effects of light in the evening and at night.”

**Figure 2.**
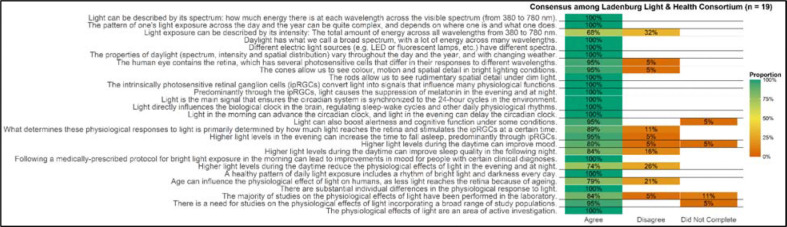
Consensus process and expert agreement levels. This figure illustrates the consensus process for 27 statements on the non-image-forming effects of light, displaying the proportion of participants (n=19) who agreed (green), disagreed (orange), or did not provide a response (orange). Horizontal bars display agreement levels for each statement, with darker shades indicating higher percentages. The consensus threshold is set at 75%. Statements that did not meet this threshold were either amended following contextual feedback or excluded from further consideration. The figure highlights areas of strong agreement as well as topics where expert opinions varied, reflecting the complexity of certain concepts in light and health research.

These statements did not meet the predefined consensus threshold, indicating variability in expert agreement regarding their formulation or interpretation (Figure 2). The coordinating committee carefully reviewed all contextual feedback provided by the Consortium. Open-ended feedback revealed that Statement 1 was considered too general, with multiple members recommending the inclusion of information on how the different characteristics of light are weighted. In response, the coordinating committee revised the statement accordingly, and it was subsequently included in the list of agreed-upon statements to be reviewed by the Consortium. In contrast, Statement 2 was deemed to lack sufficient scientific evidence and was therefore removed from the remainder of the consensus process.

Additionally, three of the original statements remained unrated by two participants, leading to missing data in the final assessment. However, even if these unrated items were categorized as “disagreed”, the affected statements would still not fall below the 75% consensus criterion. Therefore, they were retained the final set of statements (Figure 2).

### Consensus statements for public dissemination

The consensus process resulted in the development of 26 statements that were unanimously agreed upon, focusing on the effects of light on humans. These statements provide scientifically supported insights into how light influences physiological and psychological processes in addition to vision. Additionally, the consensus process involved refining these statements into simplified versions to enhance accessibility for a broader audience. Contextual information, along with relevant references, was also incorporated to provide background knowledge and real-world applications, ensuring a comprehensive understanding of the effects of light.

### Evidence rating

The quality of the supporting scientific literature was assessed using a scoring system adapted from the Oxford Centre for Evidence-Based Medicine (OCEBM) framework for rating individual studies. This modified approach allowed for consistent evaluation of the strength and reliability of the evidence cited supporting each statement, considering study design, methodological rigour, and relevance to the topic **(Supplementary Table 2).** Several statements were supported primarily by textbook knowledge or widely accepted biological principles, where empirical clinical trials are not feasible.

### Readability of statements

All scientific statements, simplified statements and contextual information were assessed for readability by an independent medical writer to ensure accessibility for a broad, non-specialist audience. The Flesch-Kincaid Grade Level for the statements was 8.6, indicating an approximate 8th-grade reading level, with a Flesch Reading Ease score of 59.3, suggesting the content is moderately easy to understand for the general public. On average, the statements contained 14.8 words per sentence and 5 characters per word. The contextual information had a Flesch-Kincaid Grade Level of 10.3 and a Reading Ease score of 43.8, reflecting a slightly more complex reading level. These sections averaged 13 words per sentence and 5.5 characters per word. In contrast, the full scientific versions of the statements were written at a Flesch-Kincaid Grade Level of 11.6, with a Reading Ease score of 42.5, indicating more advanced readability suitable for a specialist audience.

## Discussion

Effective communication of scientific knowledge to the public is essential, especially when it involves behavioural changes with significant implications for health and well-being. Translating complex scientific findings about the effects of light on health into accessible information is critical for promoting awareness and informed decision-making. This public education initiative led to the creation of 26 consensus-based statements on the effects of light on humans, designed specifically for public dissemination. A high level of agreement (>75%) was achieved for most statements among a group of scientists from different disciplines, with only two exceptions, highlighting areas requiring further future discussion and clarification. The iterative process of revising the scientific statements, simplified statements, and contextual information, in collaboration with co-authors, the consortium, and a medical writer skilled in communicating with lay audiences, helped refine the language to improve accessibility and understanding for diverse readers. This collaborative effort resulted in a measurable improvement in readability scores, ensuring that the final materials were not only scientifically accurate but also suitable for effective dissemination to the general public, educators, and policymakers. The improvement in reading level reflects a critical step toward bridging the gap between complex scientific evidence and real-world application.

### Strengths and limitations

One of the key strengths of this initiative is that it represents the first set of consensus-based statements specifically designed for public engagement on light and health. The expert-driven modified Delphi process included input from multiple disciplines, ensuring that a broad range of perspectives was incorporated. The statements underwent an iterative review and refinement process, enhancing their accuracy and clarity. Additionally, readability testing was integrated to ensure accessibility across varying levels of literacy and scientific familiarity. Collaborations with international organizations further strengthened the potential for broad dissemination and impact, increasing the likelihood that the findings will reach and influence a wide audience.

However, there are limitations to this approach. One potential limitation is the presence of biases in expert selection, as the roundtable participants came from specific disciplinary backgrounds, which may not fully capture all viewpoints. Additionally, there was a necessary trade-off between scientific precision and accessibility, leading to some degree of simplification in the messaging. Further validation of the statements is needed, including public feedback and empirical testing to assess their effectiveness in real-world communication settings. Another limitation is that the process did not include a lay audience in an iterative feedback loop. Incorporating direct input from the general public can provide valuable insights into how non-experts interpret and understand the statements, improving their clarity and impact.

Assessing the quality of evidence on the effects of light on human physiology and psychology presents unique challenges. Much of the foundational work in this field comes from foundational laboratory studies, observational research, and narrative reviews, which may not meet the highest levels of evidence grading systems such as the OCEBM Levels of Evidence we used here. Moreover, the field’s interdisciplinary nature, spanning chronobiology, psychology, lighting engineering, and public health, means diverse study designs and outcome measures are used, complicating harmonized assessment. While formal grading frameworks offer a structure for evaluation, they can undervalue high-quality mechanistic or exploratory research. Additionally, several of the statements developed here represent textbook knowledge, for which a clinical trial or similar (bio)medical study designs are unsuitable.

### Importance of public engagement in light and health

The urgency of effectively communicating the effects of light on health stems from significant changes in the modern light environment. Increased electric lighting^20^, prolonged screen exposure^21^, and a growing disconnect from natural light-dark cycles have altered human exposure patterns to light and darkness^22^.

Uncertainty persists regarding several aspects of the role of light in circadian health, sleep, and overall well-being. For instance, the consortium did not reach consensus on a statement addressing the extent to which daytime light exposure may counteract the effects of light at night, a topic that is only beginning to be explored in scientific research^23,24^.

Clear, evidence-based messaging on the health impacts of light can empower individuals to make informed choices while also guiding policymakers in shaping workplace environments, urban design, and lighting standards. Through well-structured communication, individuals and decision-makers can be encouraged to prioritize light exposure in ways that support circadian health and overall well-being.

### Plans for implementation

Building on the consensus statements and international collaborations, the next steps involve distributing the statements through organizational networks. A key milestone will be the initiative’s official launch on the UNESCO International Day of Light (16 May 2025). Further actions include (1) developing accessible outreach materials such as infographics, social media campaigns, and policy briefs to support dissemination in cooperation with public-health multipliers^17^; (2) translating statements into multiple languages to maximize accessibility and reach diverse populations; (3) establishing predefined indicators to monitor engagement and assess the impact of the communication efforts.

## Conclusion

This expert consensus delivers clear and accessible messages on the impact of light on human health. By translating scientific evidence into practical guidance, it serves as a valuable resource for public education and policy development. Public health stakeholders, including schools, employers, healthcare providers, and urban planners, can use these messages to support healthier light exposure in everyday life. The statements emphasize the need to consider light as a crucial factor in health, alongside sleep, nutrition, and physical activity.

## Supplementary Material

Supplement 1

## Figures and Tables

**Table 1:** Public health messages on the non-visual effects of ocular light exposure. The "Statement" column provides detailed descriptions using technical terminology. The "Simplified statement" column translates these concepts into more accessible language. The "Contextual information" column provides additional background and real-world examples with references to enhance comprehension and illustrate the relevance of the concepts.

Number	Statement	Simplified statement	Contextual information
1	Light can be described by its spectrum: how much energy there is at each wavelength across the visible spectrum (from approximately 380 to 780 nm).	White light is made up of multiple wavelengths, which we perceive as colours.	When we see different colours, we are actually seeing different wavelengths of light. Short wavelengths appear violet, while longer wavelengths appear indigo, blue, green, yellow, orange, and red.
2	The pattern of one's light exposure across the day and the year can be quite complex, and depends on where one is and what one does.	The amount of light around us (or the amount we are exposed to) changes as we move between indoor and outdoor spaces, throughout the day, and across seasons.	Light from the sun increases from dawn to midday and decreases in the evening. Depending on location, the amount of light in the day varies greatly between summer and winter.
3	Light exposure can be described by its intensity: The total amount of energy across all wavelengths from 380 to 780 nm, weighted according to the function of interest.	We can measure light exposure by assessing how intense the light is at different wavelengths.	We can characterise a light source by describing how much power it emits at each wavelength. This power is called intensity. Physicists measure intensity, while biological and psychological scientists study how intensity affects various processes.
4	Daylight has what we call a broad spectrum, with a lot of energy across many wavelengths.	Daylight has a wide range of energy across multiple wavelengths - it can be separated into all the colours of the rainbow.	Daylight, a combination of direct sunlight and scattered sky light, contains all visible wavelengths and more. Changes in the time of day and weather conditions shift the wavelength composition. This shift changes the colour that daylight appears.
5	Different electric light sources (e.g., LED or fluorescent lamps, etc.) have different spectra.	Different light sources generate light in different ways, producing distinct wavelengths or colour.	Different light source technologies use various materials to convert electricity into light. Different materials cause the intensity of light to spread across different wavelengths in different ways. This spread of light across wavelengths is known as spectral distribution.
6	The properties of daylight (spectrum, intensity and spatial distribution) vary throughout the day and the year, and with changing weather.	The colour, intensity, and pattern of daylight change throughout the day, with seasons, and weather.	As sunlight travels through the atmosphere, blue light is scattered more than other wavelengths. This makes the sky appear blue.
7	The human eye contains the retina, which has several photosensitive cells that differ in their responses to different wavelengths.	In the eye, the retina contains cells that allow us to detect different colours.	In the human eye, the retina contains light-sensitive cells that convert light into signals for the brain. These cells are called cones, rods, and intrinsically photosensitive retinal ganglion cells (ipRGCs)^25,26^.
8	The cones allow us to see colour, motion and spatial detail in bright lighting conditions.	Cone cells help us see colours, movement, and objects in bright light.	Cones are specialized cells in the retina. They are named for their shape (how they appear under a microscope). The highest density of cones is in the fovea (in the centre of the retina).
9	The rods allow us to see rudimentary spatial detail under dim light.	Rod cells help us see shapes and details in dim light.	Rods are highly sensitive to low light levels. They are essential for seeing at night.
10	The intrinsically photosensitive retinal ganglion cells (ipRGCs) convert light into signals that influence many physiological functions.	When ipRGCs detect light, they send signals to the brain to regulate various bodily functions.	ipRGCs send electrical signals to brain regions that regulate sleep-wake cycles, alertness, and mood.
11	Through the ipRGCs, light causes the suppression of melatonin in the evening and at night.	Light blocks the production of melatonin (a hormone that regulates sleep-wake cycles), particularly in the evening and at night.	ipRGCs express melanopsin (a light-sensitive protein). When ipRGCs detect bright light, melanopsin becomes activated. This activation triggers a neuronal pathway, blocking the production of melatonin in the pineal gland.
12	Light is the main signal that ensures the circadian system is synchronized to the 24-hour cycles in the environment.	Light is the primary signal that synchronizes the body's internal clock with the sun’s day-night cycle.	Light helps regulate the body's internal rhythms. This ensures biological processes start and stop at appropriate times.
13	Light directly influences the biological clock in the brain, regulating sleep-wake cycles and other daily physiological rhythms.	Light influences the body’s internal clock, which regulates sleep-wake patterns and other daily rhythms.	Light exposure influences sleep, hormone release, metabolism, and alertness.
14	Light in the morning can advance the circadian clock, and light in the evening can delay the circadian clock.	Morning light promotes earlier bedtime and wake time, while evening light can delay bedtime and waking.	Light tells the brain to become active. Light in the morning advances the body’s internal clock (shifts it to an earlier “time”), while light in the evening delays it (shifts it to a later “time”).
15	Light can also boost alertness and cognitive function under some conditions.	In certain situations, light can improve alertness and ability to think.	Under some conditions, exposure to high-intensity light during the day and night enhances alertness and improves cognitive performance.
16	What determines these physiological responses to light is primarily determined by how much light reaches the retina and stimulates the ipRGCs at a certain time.	The body's response to light depends on how much light and what time the light reaches the ipRGCs in the retina.	The response of ipRGCs likely depends on the time of day and circadian factors.
17	Higher light levels in the evening can increase the time to fall asleep.	Evening light exposure can make it harder to fall asleep.	Evening light exposure tells the body it is still daytime. This increases alertness and shifts the internal clock to a later time.
18	Higher light levels during the daytime can improve mood.	Bright light during the day can improve mood.	Exposure to natural daylight (the sun) or high-intensity electric light, when free from glare, can reduce stress and improve emotional balance.
19	Higher light levels during the daytime can improve sleep quality in the following night.	Exposure to more intense light during the day can improve sleep.	Exposure to higher daytime light levels reduces sleep fragmentation (awakening throughout the night) and increases deep sleep at night.
20	Following a medically-prescribed protocol for bright light, exposure in the morning can lead to improvements in mood for people with certain clinical diagnoses.	Doctors may prescribe light therapy as treatment for winter depression and other health conditions.	Exposure to bright light has been shown to improve depressive symptoms in individuals with Seasonal Affective Disorder.
21	A healthy pattern of daily light exposure includes a rhythm of bright light and darkness every day.	A healthy daily routine includes bright light during the day and darkness at night.	Maintaining a regular pattern of bright light during the day and darkness at night is associated with better physical and mental health.
22	Age can influence the physiological effect of light on humans, as less light reaches the retina because of ageing.	As people age, the lenses of the eye can become less transparent, which may reduce the effects of light on the internal clock and sleep.	With age, the eye’s lenses become less transparent, which may reduce the body’s response to light exposure.
23	There are substantial individual differences in the physiological response to light.	How people respond to light can vary greatly.	Individual differences in light sensitivity are influenced by factors such as age, genetics, and behavior.
24	The majority of studies on the physiological effects of light have been performed in the laboratory.	Most research on light’s effects on the body has been conducted in the laboratory.	Studies investigating light exposure in the real-world are needed.
25	There is a need for studies on the physiological effects of light incorporating a broad range of study populations.	More studies are needed to understand how light influences health in different groups of people.	Most studies on the physiological effects of light have focused on limited populations (specific age groups, ethnicities, or health conditions). It is important that future research studies more diverse populations over wider geographical regions.
26	The physiological effects of light are an area of active investigation.	Scientists continue to explore how light affects bodily functions and overall health.	Scientific and popular interest in the effects of light on biological rhythms, sleep, alertness, mood, and health is growing. Technological and scientific advances are enabling researchers to more precisely study different wavelengths and different intensities.

## Data Availability

The data supporting the findings of this study, including anonymized voting outcomes and finalized consensus statements, are available at https://github.com/tscnlab/SpitschanEtAl_medRxiv_2025. The data are licensed under the CC-BY 4.0 License, and the code is licensed under the MIT License. All materials used to develop public-facing statements, including simplified text and contextual explanations, are included in this manuscript and public dissemination materials. No individual-level or sensitive data were collected or analyzed as part of this consensus process.

## Appendix A: Consortium members

### Coordinating committee

**Table:**

Name	Institution	Country	ORCID
Prof. Manuel Spitschan	Technical University of Munich & Max Planck Institute for Biological Cybernetics	Germany	0000-0002-8572-9268
Dr Laura Kervezee	Leiden University Medical Center	Netherlands	0000-0002-6062-9164
Dr Oliver Stefani	Lucerne University of Applied Sciences and Arts	Switzerland	0000-0003-0199-6500
Dr Marijke Gordijn	Chrono@Work & University of Groningen	Netherlands	0000-0001-9521-8085
Dr Jennifer A. Veitch	National Research Council of Canada	Canada	0000-0003-3183-4537
Dr Renske Lok	Stanford University	USA	0000-0003-1684-5625

### Ladenburg Light and Health Consortium (in alphabetical order):

**Table:**

Name	Institution	Country	ORCID
Dr Christine Blume	University of Basel & Psychiatric Hospital of the University of Basel	Switzerland	0000-0003-2328-9612
Prof. George C. Brainard	Thomas Jefferson University	USA	0000-0003-447-25786
Dr Kai Broszio	Federal Institute for Occupational Safety and Health (BauA)	Germany	0000-0002-8269-8654
Prof. Timothy Brown	University of Manchester	United Kingdom	0000-0002-5625-4750
Jan Denneman	Good Light Group	Netherlands	0009-0006-4456-4301
Dr Maydel Fernandez-Alonso	Max Planck Institute for Biological Cybernetics	Germany	0000-0002-3179-7476
Prof. Shigekazu Higuchi	Kyushu University	Japan	0000-0001-7131-0792
Dr Daniel S. Joyce	University of Southern Queensland	Australia	0000-0002-6407-4653
Prof. Robert Lucas	University of Manchester	United Kingdom	0000-0002-1088-8029
Dr Elise M. McGlashan	University of Melbourne	Australia	0000-0002-3864-7198
Dr Raymond P. Najjar	National University of Singapore & Singapore Eye Research Institute	Singapore	0000-0002-3770-2300
Dr Luke Price	UK Health Security Agency	United Kingdom	0000-0002-4528-0566
Dr Sylvia Rabstein	Ruhr University Bochum	Germany	0000-0002-1757-847X
Dr Luc Schlangen	Eindhoven University of Technology	Netherlands	0000-0002-8424-7240
Dr David H. Sliney	IES Photobiology Committee	USA	0000-0003-4859-5982
Dr Juliëtte van Duijnhoven	Eindhoven University of Technology	Netherlands	0000-0003-1145-7033
Dr Daniela Weiskopf	Federal Office for Radiation Protection	Germany	0009-0001-8637-7911
Prof. Kenneth Wright	University of Colorado Boulder	USA	0000-0002-1781-7469
Dr Johannes Zauner	Technical University of Munich & Max Planck Institute for Biological Cybernetics	Germany	0000-0003-2171-4566
