# Supplementary material for: Evidence-based public health messaging on the non-visual effects of ocular light exposure: A modified Delphi expert consensus
Source: medRxiv. 2025 May 8:2025.05.07.25327160. Preprint. [Version 1] doi: 10.1101/2025.05.07.25327160 (PMC12083622; doi:10.1101/2025.05.07.25327160)
Supplement: Supplementary file 1 [file NIHPP2025.05.07.25327160v1-supplement-1.pdf]

medRxiv preprint doi: https://doi.org/10.1101/2025.05.07.25327160; this version posted May 8, 2025. The copyright holder for this preprint (which was not certified by peer review) is the author/funder, who has granted medRxiv a license to display the preprint in perpetuity.
It is made available under a CC-BY 4.0 International license .

Supplementary Table 1: Rating of the quality of evidence. This table lists each reference, corresponding number, general study type, and an evidence quality rating. Ratings are based on a modified version of the Oxford Centre for Evidence-Based Medicine levels for individual studies. The five levels of evidence are defined as follows: (1) Properly powered and conducted randomized clinical trial or systematic review with meta-analysis; (2) Well-designed controlled trial without randomization or prospective comparative cohort trial; (3) Case-control studies or retrospective cohort study; (4) Case series with or without intervention or cross-sectional study; (5) Opinion of respected authorities or case reports. As many of the statements represent textbook knowledge of basic physics and biology, we have added the rating category (–) for these statements.

Number	Statement	Main supporting sources	Rating
1	Light can be described by its spectrum: how much energy there is at each wavelength across the visible spectrum (from approximately 380 to 780 nm).	[27,28]	–
2	The pattern of one's light exposure across the day and the year can be quite complex, and depends on where one is and what one does.	[29]	–
3	Light exposure can be described by its intensity: The total amount of energy across all wavelengths from 380 to 780 nm, weighted according to the function of interest.	[30]	–
4	Daylight has what we call a broad spectrum, with a lot of energy across many wavelengths.	[28]	–
5	Different electric light sources (e.g., LED or fluorescent lamps, etc.) have different spectra.	[31,32]	–
6	The properties of daylight (spectrum, intensity and spatial distribution) vary throughout the day and the year, and with changing weather.	[32-35]	–
7	The human eye contains the retina, which has several photosensitive cells that differ in their responses to different wavelengths.		–
8	The cones allow us to see colour, motion and spatial detail in bright lighting conditions.	[36,37]	–
9	The rods allow us to see rudimentary spatial detail under dim light.	[38,39]	–
10	The intrinsically photosensitive retinal ganglion cells (ipRGCs) convert light into signals that influence many physiological functions.	[25,40,41]	–
11	Through the ipRGCs, light causes the suppression of melatonin in the evening and at night.	[42-44]	1
12	Light is the main signal that ensures the circadian system is synchronized to the 24-hour cycles in the environment.	[2,4,22,45]	2
13	Light directly influences the biological clock in the brain, regulating sleep-wake cycles and other daily physiological rhythms.	[2,4,22]	2
14	Light in the morning can advance the circadian clock, and light in the evening can delay the circadian clock.	[6,7]	2
15	Light can also boost alertness and cognitive function under some conditions.	[8,9,12]	2
16	What determines these physiological responses to light is	[44,46-49]	2

medRxiv preprint doi: https://doi.org/10.1101/2025.05.07.25327160; this version posted May 8, 2025. The copyright holder for this preprint (which was not certified by peer review) is the author/funder, who has granted medRxiv a license to display the preprint in perpetuity.
It is made available under a CC-BY 4.0 International license .

	primarily determined by how much light reaches the retina and stimulates the ipRGCs at a certain time.		
17	Higher light levels in the evening can increase the time to fall asleep.	[49-53]	1
18	Higher light levels during the daytime can improve mood.	[23,54]	3
19	Higher light levels during the daytime can improve sleep quality in the following night.	[55-57]	2
20	Following a medically-prescribed protocol for bright light, exposure in the morning can lead to improvements in mood for people with certain clinical diagnoses.	[58,59]	1
21	A healthy pattern of daily light exposure includes a rhythm of bright light and darkness every day.	[15,24,60,61]	3
22	Age can influence the physiological effect of light on humans, as less light reaches the retina because of ageing.	[62-65]	3
23	There are substantial individual differences in the physiological response to light.	[53,66-68]	2
24	The majority of studies on the physiological effects of light have been performed in the laboratory.	[69]	5
25	There is a need for studies on the physiological effects of light incorporating a broad range of study populations.	[70,71]	5
26	The physiological effects of light are an area of active investigation.	[72]	5
